# Supplementary material for: A bioavailable 87Sr/86Sr isoscape of Mongolia: Implications for the reconstruction of past human and animal mobility
Source: PLoS One. 2025 Dec 3;20(12):e0336327. doi: 10.1371/journal.pone.0336327 (PMC12674565; doi:10.1371/journal.pone.0336327)
Supplement: S3 File — (PDF) [file pone.0336327.s003.pdf]

A bioavailable $^{87}Sr/^{86}Sr$ isoscape of Mongolia: implications for the reconstruction of past human and animal mobility

Mael Le Corre[1,2], Eric Douville[3], Arnaud Dapoigny[3], Khanh-Vi Tran[3], Ganbold Enkhbayar[4], Tsagaan Turbat[5], Sébastien Lepetz[1], Antoine Zazzo[1]

[1] BioArchéologie, Interactions Sociétés Environnements (BioArch, UMR 7209), Muséum National d'Histoire Naturelle, Sorbonne Université, Centre National de la Recherche Scientifique (CNRS), CP 56, 55 rue Buffon, 75005 Paris, France.

[2] Laboratoire de Géologie de Lyon, Terre, Planètes, Environnement (LGLTPE, UMR 5276), École Normale Supérieure Lyon, Université Lyon 1, Centre National de la Recherche Scientifique (CNRS), 46 Allée d'Italie, 69342 Lyon Cedex 07, France.

[3] Laboratoire des Sciences du Climat et de l'Environnement (LSCE/IPSL, UMR 8212), Laboratoire des Sciences du Climat et de l'Environnement, LSCE/IPSL, UMR 8212 CEA-CNRS-UVSQ, Université Paris-Saclay, F-91191 Gif-sur-Yvette, France.

[4] National Museum of Mongolia, Juulchin Street-1, Chingeltei District, 15160 Ulaanbaatar, Mongolia.

[5] Institute of Nomadic Archaeology and Department of Anthropology and Archaeology, National University of Mongolia, Ikh Surguuli Street 1, 14200 Ulaanbaatar, Mongolia.

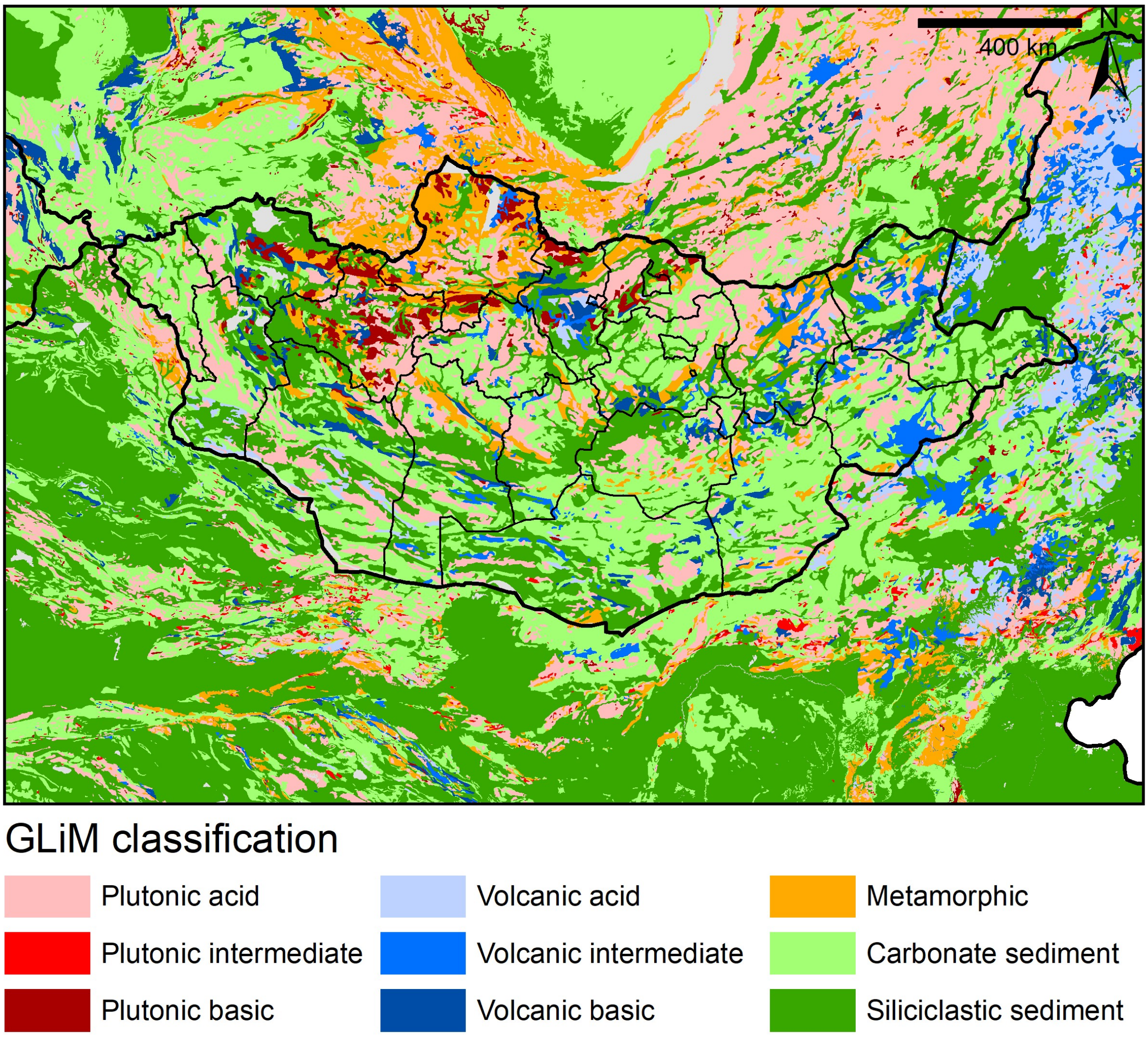

Fig S1. Geological map of Mongolia based on the GLiM (Global Lithological Map) classification (Hartmann & Moosdorf, 2012).

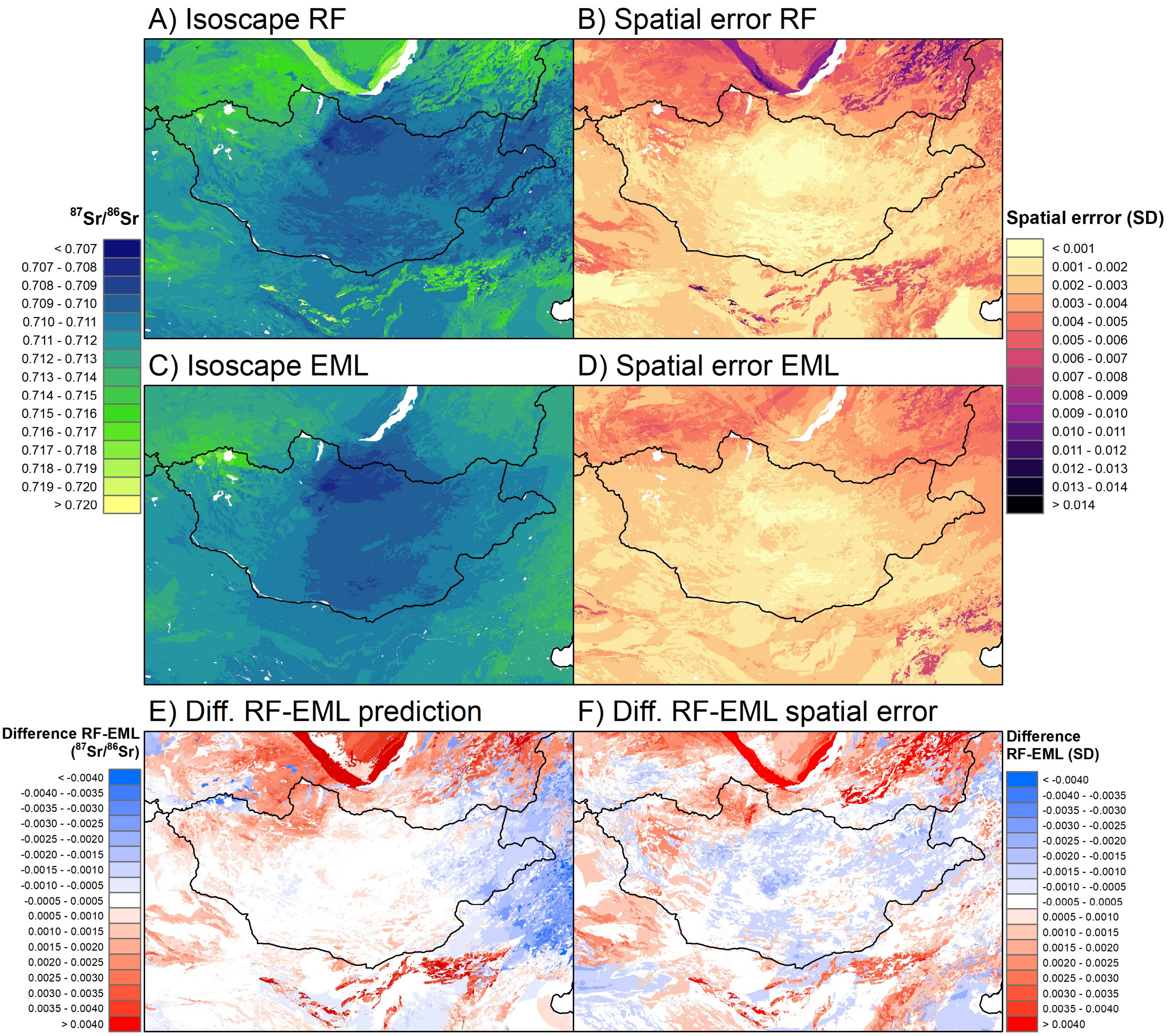

Fig S2. Comparison between the RF and the EML bioavailable $^{87}Sr/^{86}Sr$ isoscapes. (A) RF bioavailable $^{87}Sr/^{86}Sr$ isoscapes with (B) the associated spatial uncertainty. (C) EML bioavailable $^{87}Sr/^{86}Sr$ isoscapes with (D) the associated spatial uncertainty. Differences (E) between the RF and EML isoscapes (RF – EML) and (F) between the RF and EML spatial uncertainty maps (RF - EML). Negative values, in blue, indicate that prediction and spatial error of the EML are higher than the prediction and spatial error of the RF.

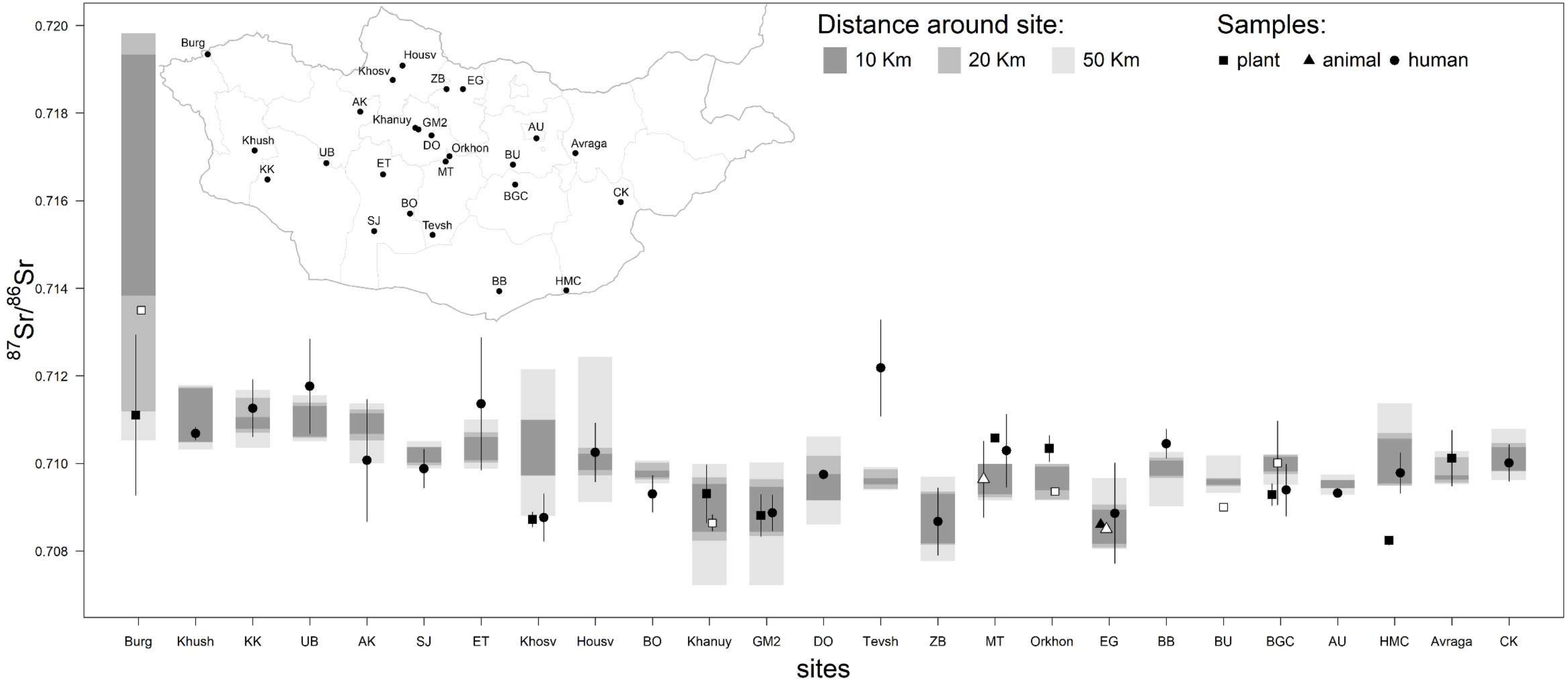

Fig S3. Comparison between $^{87}Sr/^{86}Sr$ values of modern samples (plant, animals) and archaeological remains (human, animals) collected at 25 sites across Mongolia and the range of bioavailable $^{87}Sr/^{86}Sr$ predicted by the EML isoscape within 10 km, 20 km and 50 km of the archaeological sites. Filled and open symbols correspond to archaeological and modern data respectively.The map shows the locations of the sites across Mongolia. Avraga: Avraga, AU: Agui Uul, AK: Avdalai Khyasaa, BGC: Baga Gazaryn Chuluu, Burg: Burgast, BB: Bayanbulag, BO: Bor Ovoo, CK: Chandman Khar, DO: Dunde Orontso cemetery, Egiin Gol, ET: Emeelt Tolgoi, GM2: Gold Mod 2, HMC: Hets Mountain cave, Houvs: Houvsgol, Khanuy: Khanuy valley, Khosv: Khosvgol (A, B, C, D, F), Khush: Khushuut, Khyar: Khyar Kharaach, MT: Maikhan Tolgoi, Orkhon: Orkhon valley (Moiltyn Am, Orkhon 1,7), SJ: Shine Jinst, Tevsh: Tevsh, UB: Ulaan Boom, ZB: Zuun Bel.

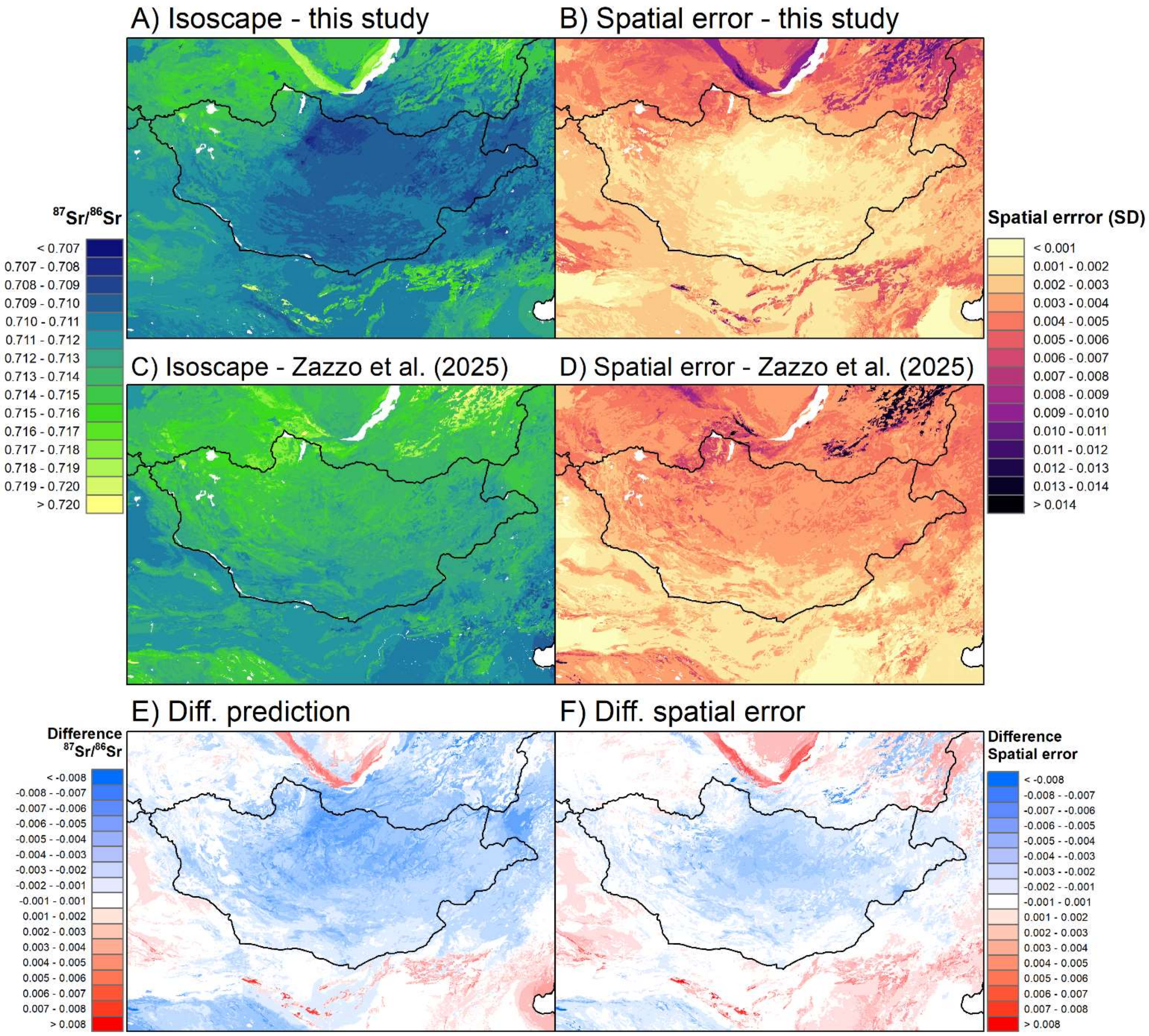

Fig S2. Comparison with the bioavailable Altai-calibrated $^{87}Sr/^{86}Sr$ isoscape from Zazzo et al. (2025). (A) RF bioavailable $^{87}Sr/^{86}Sr$ isoscape with (B) the associated spatial uncertainty. (C) Altai-calibrated bioavailable $^{87}Sr/^{86}Sr$ isoscape (2025) with (D) the associated spatial uncertainty. Differences between the prediction (E) and the spatial uncertainty (F) of the present study and the Altai-calibrated isoscape. Negative values, in blue, indicate that prediction and spatial error of the Altai-calibrated isoscape are higher than the prediction and spatial error of the present study. Basemaps for (C) and (D) adapted from Zazzo et al. (2025)

References :

Hartmann, J., & Moosdorf, N. (2012). The new global lithological map database GLiM: A representation of rock properties at the Earth surface. *Geochemistry, Geophysics, Geosystems*, *13*(12). https://doi.org/10.1029/2012GC004370.

Zazzo, A., Le Corre, M., Lazzerini, N., Marchina, C., Bayarkhuu, N., Bernard, V., Cervel, M., Fiorillo, D., Joly, D., Thil, F., Turbat, T., Balter, V., Coulon, A., & Lepetz, S. (2025). 3000 yr-old patterns of mobile pastoralism revealed by multiple isotopes and radiocarbon dating of ancient horses from the Mongolian Altai. *PLoS ONE* 20(5): e0322431. https://doi.org/10.1371/journal.pone.0322431.
